# Advanced gallbladder cancer accompanied with cancer-associated dermatomyositis: A case report and literature review

**DOI:** 10.1097/MD.0000000000029477

**Published:** 2022-07-08

**Authors:** Haruka Kuroda, Atsushi Yamaguchi, Shuhei Sugata, Takuro Hamada, Riho Moriuchi, Kaoru Wada, Yuzuru Tamaru, Ryusaku Kusunoki, Toshio Kuwai, Hirotaka Kouno, Takashi Kurashige, Tsuyoshi Torii, Akihisa Saito, Kazuya Kuraoka, Hiroshi Kohno

**Affiliations:** a Department of Gastroenterology, National Hospital Organization Kure Medical Center and Chugoku Cancer Center, Kure, Hiroshima, Japan; b Department of Neurology, National Hospital Organization Kure Medical Center and Chugoku Cancer Center, Kure, Hiroshima, Japan; c Department of Pathology, National Hospital Organization Kure Medical Center and Chugoku Cancer Center, Kure, Hiroshima Prefecture, Japan.

**Keywords:** adenocarcinoma, gallbladder, gallbladder cancer, cancer-associated dermatomyositis (CADM), dermatomyositis, polymyositis

## Abstract

**Rationale:**

Muscle weakness due to cancer-associated dermatomyositis (CADM) can be misdiagnosed as cancer cachexia and disuse atrophy.

**Patient concerns:**

A 75-year-old female was admitted to our institute with muscle weakness, dysphagia, and suspected gallbladder cancer. Computed tomography and cytopathological examinations of the liver biopsy and fine-needle aspiration from swollen lymph nodes using endoscopic ultrasonography revealed cancer in the gallbladder body and metastasis to the lymph nodes around the abdominal aorta. We avoided the administration of anticancer drugs due to her poor general condition.

**Diagnosis:**

Subsequently, we diagnosed her with muscle weakness and dysphagia as a result of CADM using species from muscle and skin biopsy.

**Interventions and Outcomes:**

Prednisolone therapy and anticancer agents partially improved the patient symptoms.

**Lessons:**

CADM is reported to be associated with a high incidence of dysphagia, which may aid in the diagnosis of this disease.

## 1. Introduction

Dermatomyositis (DM) is an autoimmune disease characterized by muscle inflammation, unique skin lesions, and positive autoantibodies. Adult cases of DM have been reported to accompany malignancies at frequencies of 10%−30%.^[1]^ The malignancies with which DM is most commonly associated are ovarian and lung cancers,^[2]^ while there are few reported cases of gallbladder cancer. Treatments for DM usually involve a combination of immunosuppressive drugs and anticancer therapy,^[3]^ in which anticancer therapy may lead to the resolution of DM. In this instance, we encountered a case of gallbladder cancer with cancer-associated dermatomyositis (CADM), which was difficult to differentiate from cancer cachexia and disuse atrophy.

## 2. Case report

**Figure 1.**
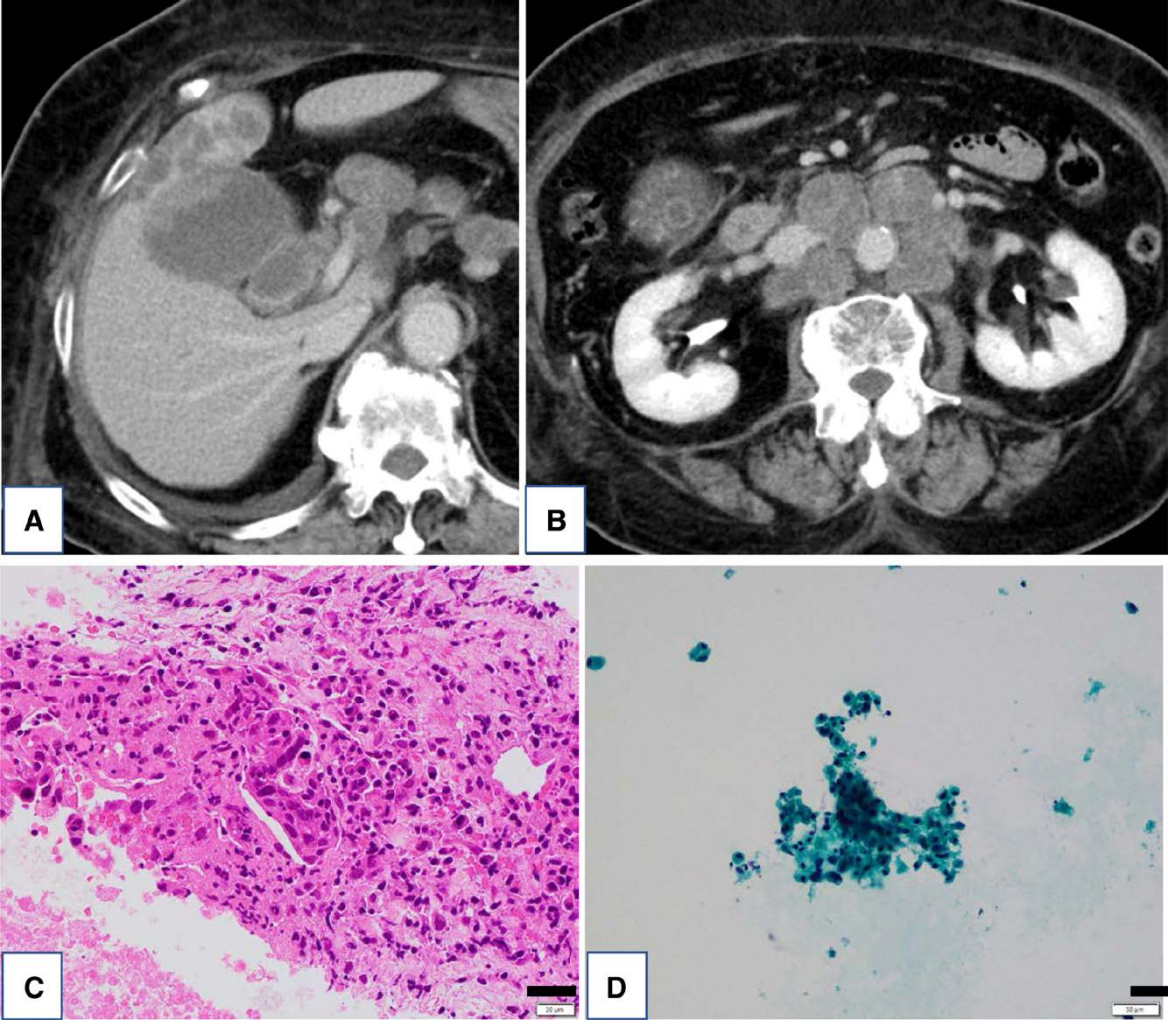
Abdominal CT images and cytopathological examination. There is a 4 × 1.5 cm mass in the body of the gallbladder with direct infiltration to segment 4 of the liver (A). Multiple lymph nodes around the abdominal aorta are swollen (B). Poorly differentiated adenocarcinoma is evident on cytological examination of species obtained from liver biopsy (C). Poorly differentiated adenocarcinoma is also observed on cytological examination of species obtained from a fine-needle aspiration using endoscopic ultrasonography (D). Scale bars: 50 µm (C), 20 µm (D).

**Figure 2.**
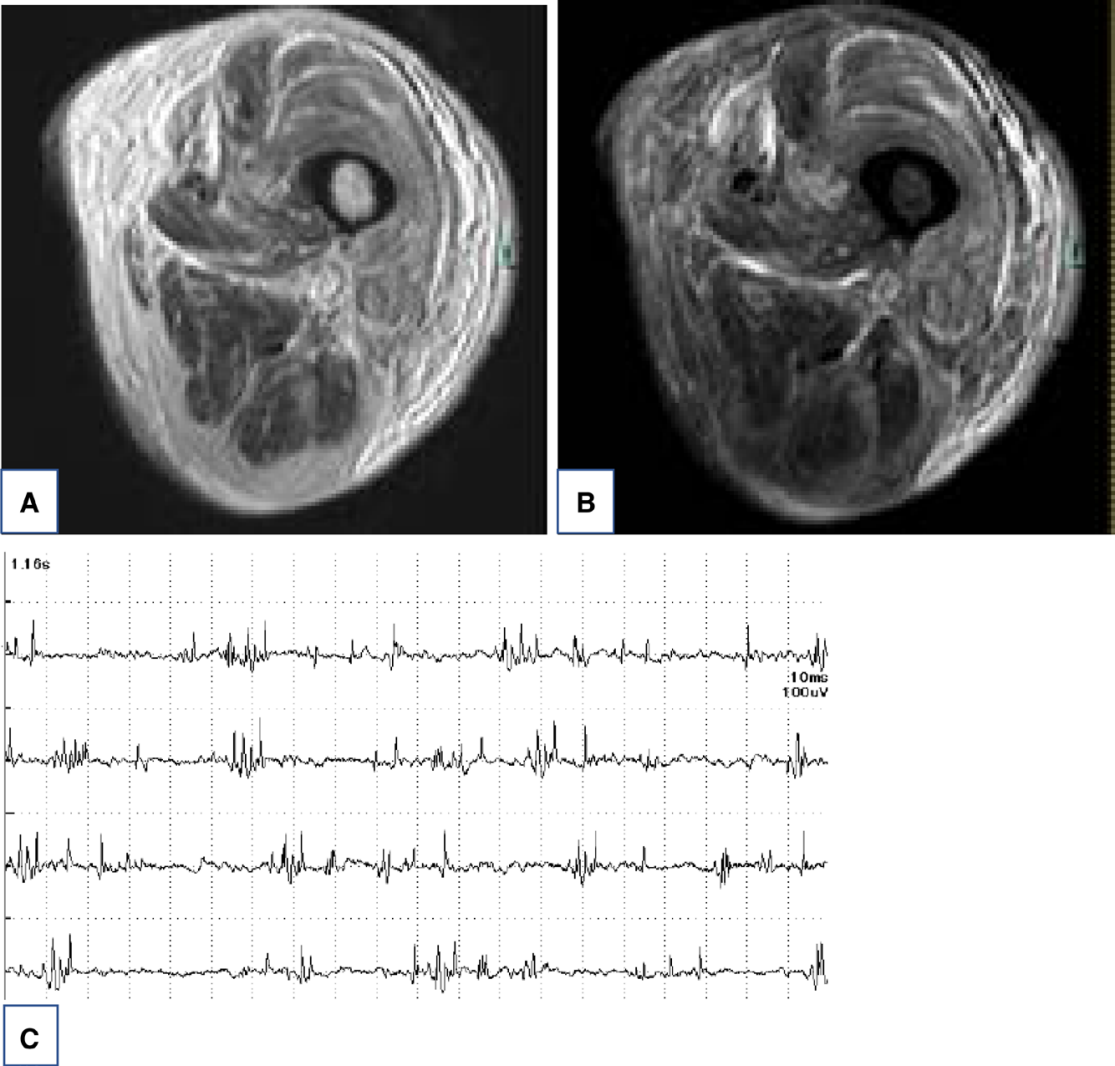
Magnetic resonance imaging of the left thigh showed a high signal intensity in T2-weighted and (A) short TI inversion recovery images (B). The needle electromyogram shows early recruitment and motor unit potential with low amplitude and polyphase (C).

**Figure 3.**
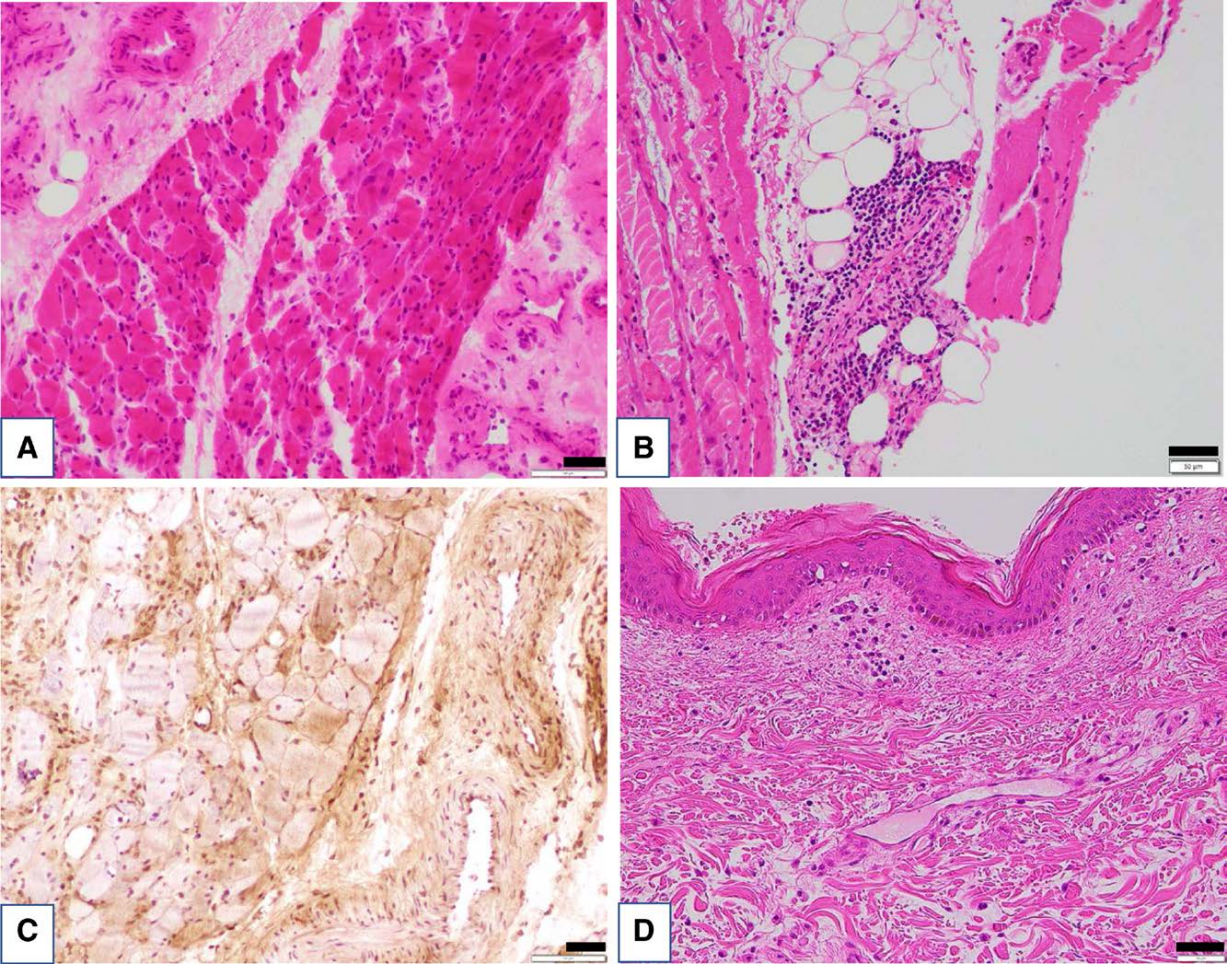
Muscle biopsy specimens from the left biceps brachii muscle show perifascicular atrophy (A), perivascular cuffing of inflammatory cells (B), and perifascicular expression of myxovirus resistance protein A (C). Specimens of the skin around the biceps muscles show mild epidermal atrophy, hydropic degeneration of the basal cells, sparse inflammatory infiltrate accentuating superficial dermal vessels, and mucin present in the dermis (D). Scale bars: 100 µm (A, C) and 50 µm (B, D).

**Figure 4.**
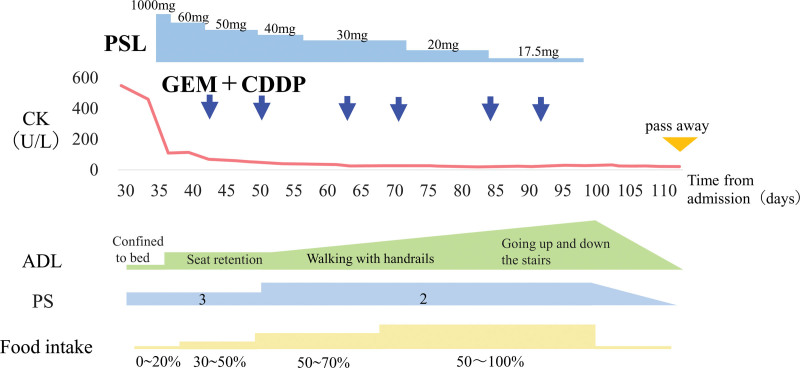
Clinical course and changes in CK serum levels, activities of daily living (ADL), performance status (PS), and food intake. After prednisolone administration and anticancer therapy, CK rapidly normalized while ADL, PS, and food intake gradually improved. PSL, prednisolone; GEM + CDDP, gemcitabine plus cisplatin.

A 75-year-old female was admitted to her previous hospital in October 2019 with complaints of general fatigue, muscle weakness, and dysphagia. Her symptoms worsened, and gallbladder cancer was identified by abdominal computed tomography (CT). She was hospitalized at our institute in November 2019. The patient was being treated for hypertension and diabetes mellitus. Her physical findings were as follows: height, 149 cm; body weight, 67.7 kg; body temperature, 36.9 °C; blood pressure, 129/62 mm Hg; and pulse rate, 74 beats/min. Her breathing and heart sounds were within normal limits. Characteristic erythematous rashes, such as Gottron papules, heliotrope rash, shawl sign, and V neck signs, were not observed. A manual muscle test scored fairly for the proximal muscles of the extremities. Laboratory data showed elevated serum levels of creatine kinase (CK) (2342 U/L), C-reactive protein (7.69 mg/dL), aspartate aminotransferase (160 U/L), alanine aminotransferase (57 U/L), lactate dehydrogenase (569 U/L), and γ-glutamyl transpeptidase (76 U/L). The serum levels of carcinoembryonic antigen and carbohydrate antigen 19-9 were normal. Abdominal CT revealed a 4 × 1.5 cm mass in the body of the gallbladder, which directly infiltrated segment 4 of the liver; moreover, multiple lymph nodes around the abdominal aorta were swollen (Fig. 1A, B). Specimens from a liver biopsy (Fig. 1C) and fine-needle aspiration from lymph nodes using endoscopic ultrasonography (Fig. 1D) showed poorly differentiated adenocarcinomas, and we diagnosed the patient with gallbladder cancer with multiple lymph node metastases. We prioritized nutritional therapy and rehabilitation over anticancer therapy due to the poor performance status (PS) (PS3). CK decreased spontaneously; therefore, we suspected that the patient’s poor general condition and muscle weakness were due to cancer cachexia. Subsequently, she continued nutritional therapy and rehabilitation but showed no improvement in PS; consequently, we decided to conduct deep examinations considering neurological and muscle diseases. Magnetic resonance imaging of the thigh and upper arm showed a high signal intensity on T2-weighted images and short TI inversion recovery images (Fig. 2A, B). In addition, needle electromyography showed early recruitment and motor unit potential with low amplitude and polyphase (Fig. 2C). Together, these 2 findings suggest myositis. Based on these results, we suspected the patient had DM and measured autoantibodies specific to DM (Table 1), which yielded positive results for the antitranscriptional intermediary factor 1-γ (TIF1-γ) antibody, which is particularly positive in DM associated with malignancy. Muscle biopsy specimens from the left biceps brachii muscle showed perifascicular atrophy (Fig. 3A), perivascular cuffing (Fig. 3B), and perifascicular expression of myxovirus resistance protein A (Fig. 3C); furthermore, the skin biopsy specimen from the left arm showed epidermal atrophy, hydropic degeneration of basal cells, sparse inflammatory infiltrate accentuating superficial dermal vessels, mucin present in the dermis (Fig. 3D). These findings are consistent with those for DM. We diagnosed this case as CADM. After diagnosis, the patient was treated with methylprednisolone (1000 mg/day) for 3 days, and the dosage was tapered from 60 mg. In addition, anticancer therapy with gemcitabine plus cisplatin was administered to treat gallbladder cancer. Consequently, CK quickly normalized, and PS, food intake, and appetite gradually improved. However, she developed pneumonia, her general condition deteriorated rapidly, and she died (Fig. 4).

**Table 1.** Result of measured autoantibody associated with dermatomyositis.

AntiARS antibody	<5.0 (reference <25.0)
antiMi2 antibody	<5.0 (reference <53)
antiTIF1γ antibody	128 (reference <32)
Anti-MDA5antibody	<7.0 (reference <32)

ARS = aminoacyl-tRNA synthetase, MDA5 = melanoma differentiation-associated gene 5, TIF1γ = transcriptional intermediary factor 1 γ.

## 3. Discussion

DM is an autoimmune disease that causes proximal muscle weakness over the course of several weeks to months. It has been reported that approximately 10%−30% of adult cases accompany malignant tumors.^[1]^ When treating CADM, it is important to treat the malignant tumor itself.^[3]^ In our case, steroid therapy and chemotherapy, which were not administered to the patient at the first diagnosis, yielded a clear improvement in symptoms. Therefore, we regretted the delayed diagnosis of CADM and suspected that an earlier diagnosis might have improved her general condition and survival.

We collected from PubMed 6 case reports of CADM associated with gallbladder cancer between 1960 and 2020 using the keywords: “dermatomyositis” and “gallbladder cancer,” as well as 2 cases from *Igakutyuozassi* (a Japanese database) between 1990 and 2020 using the keywords: “*tannougan*” and “*hifukinen.*” We reviewed these 8 cases and our own (Table 2).^[4–11]^ All patients were females, and the median age was relatively high (72 years). Therefore, CADM should be carefully differentiated from age-related disuse syndrome and cancer cachexia. Although serum CK levels varied, the patient consistently showed above-normal levels. Therefore, CADM should be considered when patients with malignancies have high serum CK levels. In addition, there have been reported cases in which serum CK levels decreased due to remaining in bed after admission,^[12]^ and care should be taken when considering patients without upregulation of CK.

**Table 2.** Nine cases of gallbladder cancer accompanied with cancer-associated dermatomyositis.

Author	Reported year	Age	Sex	Clinical stage	CK	Skin Findings	Muscle findings	Treatment	Outcome	Prognosis	Ref.
					(IU/ml)				in dermatomyositis	in gallbladder cancer	
Lewis et al	1962	72	F	unknown	unknown	Skin rash on both upper extremities and chest	Muscle weakness in proximal muscles	PSL	Unknown	Dead	^[4]^
							dysphagia				
Futei et al	1994	77	F	III	unknown	Skin rash on the face, trunk, and extremities	Muscle weakness in proximal muscles	PSL	Improvement	Dead	^[5]^
						Gottron papules			(mild)		
Takeda et al	1996	63	F	III	unknown	Generalized skin rash	Muscle tenderness in proximal muscles	PSL	Unknown	Dead from another cause	^[6]^
								Chemoradiation			
Yiannopoulos et al	2002	75	F	III	350	Gottron papules	Muscle weakness in neck and shoulder	PSL Cholecystectomy	Improvement	Dead	^[7]^
						Heliotrope rashes	dysphagia		(mild)		
Kundu et al	2005	44	F	III	1659	Skin rash on Face and Neck	General fatigue	PSL	Improvement	Unknown	^[8]^
							Muscle tenderness		(mild)		
Babac et al	2013	68	F	II	22,250	none	Muscle tenderness and weakness in proximal muscles	PSL	Complete recovery	Alive	^[9]^
								Cholecystectomy			
Sawada et al	2014	90	F	III	7811	Gottron papules	Muscle weakness in proximal muscles	PSL	Improvement	Dead	^[10]^
						Heliotrope rashes			(mild)		
Petta et al	2015	48	F	IV	308	Skin rash on Face and ears	Muscle tenderness in proximal muscles	PSL	aggravation	Dead	^[11]^
							dysphagia	Gemcitabine plus cisplatin			
Our case	2021	75	F	IV	2342	none	Muscle tenderness in proximal muscles	PSL	Improvement	Dead	
							dysphagia	Gemcitabine plus cisplatin	(mild)		

CK = serum level of creatine kinase, F = female, M = male, PSL = prednisolone, Ref. = Reference number.

Our patient had no characteristic DM-associated erythematous rash, such as Gottron papules or heliotrope rash, but we diagnosed her with DM pathologically. Retrospectively, she had reddening of the skin, unlike Gottron papules and heliotrope rash, which was noted by her previous doctor. Furthermore, of the 9 patients in our literature review, only 2 exhibited heliotrope rash and 3 Gottron papules, and there was 1 case without even any reddening of the skin. Given these observations, DM should be considered when patients with malignancy have muscle weakness, even if they do not have obvious reddening of the skin and increased serum CK levels.

All patients underwent prednisolone therapy, 2 underwent cholecystectomy, and 3 received anticancer drug therapy. Symptoms worsened in 1 patient, were downregulated in 5, and improved completely in 1. The only patient in complete remission underwent radical surgery for gallbladder cancer. We tentatively conclude that cancer treatment is the most important factor for controlling DM.

In our literature review, proximal muscle weakness was observed in all patients and dysphagia was observed in 44% (4/9). This frequency was relatively high compared to all patients with DM (the reported association of dysphagia is 12%−60%^[13]^). In addition, antiTIF1-γ antibody (which was positive in our case) has been reported to be associated with malignancy.^[14]^ TIF1-γ is a transcriptional intermediate factor that suppresses tumor growth; therefore, antibodies against TIF1-γ might induce an antigen-antibody reaction to regenerate myocytes, resulting in myositis.^[15,16]^ Recently, antiTIF1-γ antibodies have been reported to be associated with dysphagia and have attracted much attention.^[17,18]^ Patients with DM with or without dysphagia are positive for antiTIF1-γ antibodies at frequencies of 71% and 19%, respectively.^[14,17,18]^ Although the mechanism of frequent dysphagia in patients with antiTIF1-γ antibodies is unclear, the presence of dysphagia and positivity for antiTIF1-γ antibodies might be useful for the diagnosis of CADM.

## 4. Conclusions

Here, we report a case of CADM in which the diagnosis was delayed. This disease should be considered when performing medical treatment for patients with cancer with muscle weakness following dysphagia, particularly in the neck. Furthermore, the measurement of antiTIF1-γ antibodies might be helpful for the diagnosis of CADM.

### Author contributions

Conceptualization: Haruka Kuroda, Atsushi Yamaguchi.

Data curation: Haruka Kuroda, Atsushi Yamaguchi, Shuhei Sugata, Takuro Hamada, Riho Moriuchi, Kaoru Wada.

Writing: Haruka Kuroda, Atsushi Yamaguchi.

Writing-review: Atsushi Yamaguchi, Toshio Kuwai, Hirotaka Kouno, Hiroshi Kohno, Takashi Kurashige, Tsuyoshi Torii.

Pathological diagnosis: Takashi Kurashige, Akihisa Saito, Kazuya Kuraoka.
